# Problematic features of episode-based definitions of depression and a preliminary proposal for their replacement

**DOI:** 10.3389/fpsyt.2023.1121524

**Published:** 2023-03-15

**Authors:** Scott B. Patten

**Affiliations:** Departments of Psychiatry and Community Health Sciences, University of Calgary, Calgary, AB, Canada

**Keywords:** depression, depressive disorders, classification, measurement, assessment

## Abstract

Episodes of depression are constructed by imposing temporal and symptom-severity thresholds onto symptom levels that vary over time, resulting in a loss of information. Consequently, it is widely acknowledged that binary categorization of depressive episodes is problematic. Binary classification can make similar symptom levels appear different and different symptom levels appear similar. Furthermore, symptom severity is only one of several thresholds that are applied in the construction of depressive episodes in DSM-5 and ICD-11, others being: a minimum duration of symptoms, the application of a “no significant symptoms” threshold for remission, and time requirements (e.g., 2  months) for remission. Application of each of these thresholds leads to a loss of information. The joint occurrence of these four thresholds creates a complex set of circumstances in which similar patterns of symptoms may be categorized differently and different patterns may be categorized as similar. The ICD-11 definition can be expected to lead to better classification than the DSM-5 approach since it does not require two symptom-free months for remission, eliminating one of four problematic thresholds. A more radical change would be to adopt a truly dimensional perspective which would need to incorporate new elements to reflect time spent at various levels of depression. Such an approach, however, seems feasible both in clinical practice and research.

## 1. Categories versus dimensions of depression

Episodes currently form the foundation of diagnostic definitions of depression. For example, in DSM-5, the core feature of Major Depressive Disorder (MDD) is the occurrence of one or more major depressive episodes (MDE) occurring in the absence of a history of manic or hypomanic episodes (1). Definitions in ICD-11 are similar to those of DSM-5 (2).

The potential advantage of dimensional measurement is avoidance of the loss of information that occurs when any inherently dimensional variable is categorized using a threshold (3). This issue is evident when screening scales that measure the symptom-based ‘A’ criterion for MDE, such as the PHQ-9 (4, 5) are considered. The distribution of PHQ-9 ratings shows no discontinuity around the presumed interpretive threshold of 10, such that application of the cut-point seems arbitrary (6). Although diagnostic criteria are not based on a straightforward numerical threshold (such as the cut point on a screening scale), they are nevertheless based on thresholds. The ‘A’ criterion in DSM-5 lists nine symptoms of which at 5 must be present for a diagnosis of Major Depressive Episode (MDE) is to be made (1). This creates a *de facto* threshold for symptom expression operationalized as a symptom count. The symptom-based features of a depressive episode in ICD-11 are very similar, except that hopelessness and worthlessness are viewed as different features rather than being included together as one of nine specified symptoms in DSM-5 (2). Both definitions require that at least one of the symptoms be either depressed mood or loss of interest (what ICD-11 calls the “affective cluster” of symptoms), although one study has reported that this requirement has little impact on diagnostic frequencies arising from application of the criteria (7).

The PHQ-9 (4, 5) has nine items roughly aligning with the 9 symptoms listed in the ‘A’ criterion of the DSM definition. If a threshold of 10+ is used to interpret PHQ-9 ratings as indicative of clinically significant depression, then nearly identical scores of 9 and 10 are interpreted as representing distinct categories, whereas very different scores such as 10 and 27, or 0 and 9 are interpreted as being similar. This illustrates the loss of information that occurs with categorization. The same issue emerges from the less explicit threshold of symptom severity built into the DSM-5 and ICD-11 definitions of depressive episodes.

The distinction between depression as a category and depression as a dimension was discussed over 20 years ago by Goldberg, who contrasted two ways of thinking about the problem: a belief that disorders have a real existence, but are not directly measureable (what Goldberg called a “Platonic” approach) and an “Aristotelian” approach in which symptoms are considered real in themselves (8). In the Platonic approach, people with higher symptom levels could be understood as being more likely to have the unseen underlying pathology. From what Goldberg called the Aristotelian perspective, the symptoms themselves represent the reality of depression, and dividing them into categories merely simplifies their interpretation. These ideas were further developed by Andrews et al. (6), who characterized the “real but unmeasurable” concept as that of a latent characteristic that could be studied using statistical methods capable of modeling latent variables. They posited that this latent characteristic would be a dimension. Both Goldberg & Andrews et al. thereby adopted a preference for dimensional measurement, while also acknowledging the clinical utility of diagnostic classification. Other authors have criticized the idea that categories have clinical utility (3), articulating an even stronger preference for dimensions. Application of diagnostic criteria can be viewed as an imperfect measurement strategy, something akin to a diagnostic test that provides helpful information but has imperfect sensitivity and specificity. Short of arguing that diagnostic categories be abandoned, Andrews et al. expressed optimism that dimensional scales such as the PHQ-9 could supplement diagnostic categorization, an idea that may be coming to fruition as Section III of DSM-5 includes a cross-cutting instrument for assessing symptoms that, in the case of depression, uses items similar to those of the PHQ-9 (1).

## 2. Other thresholds built into the DSM-5 definition

While previous authors have commented on the advantages of dimensional symptom severity measures over categorical ones, less attention has been given to the existence of additional dimensions and thresholds that also contribute to the construction of episodes in the DSM-5 and ICD-11 diagnostic systems. In fact, the DSM-5 definition of MDE involves the application of three additional thresholds (and ICD-11 applies an additional two) to inherently dimensional variables in its definition of MDE. Application of each of these thresholds can also contribute to a loss of information, compounding and magnifying the threshold-related problems that occur during the construction of episodes.

Two of the additional thresholds are time thresholds, one of which specifies that symptoms must persist for at least 2 weeks before a diagnosis can be made. An episode lasting 13 days, through the application of this threshold, would seem different than one lasting 14 days and an episode lasting 14 days would be categorized similarly to one lasting one year, such that the familiar loss of information occurs. This issue has not been a source of controversy, perhaps because clinicians inevitably see patients that have been experiencing symptoms for much longer than two weeks or treat patients at high risk of relapse, where change in mood over such a brief interval may be clinically important. The duration threshold does however produce problems for psychiatric epidemiology. In community populations, the modal duration of MDE has been reported to be 2 weeks, with longer episodes occurring at a progressively diminishing frequency (9), analogous to what is seen with symptom severity measures. This raises questions about the validity of diagnostic criteria and likely leads to exaggerated estimates of the community prevalence of clinically significant depressive disorders (10, 11).

Other issues emerge from the definition of when an episode ends. The threshold used by DSM-5 is that there should be no significant symptom for at least two months in order to declare an episode in remission (1). This requires application of two thresholds. The first part of this definition involves a threshold of “no significant symptoms.” This threshold can be difficult to define precisely. Mild or transient depressive symptoms often occur in healthy people and various levels of symptoms may be significant for some but not all people due their associated levels of distress or dysfunction as well as other factors such as interpersonal differences in tolerating distress. Whether the symptoms cause functional impairment is an important consideration, but also depends on a person’s coping strategies, the effectiveness of these strategies, and what sort of demands a person faces. With the PHQ-9, scores <5 have come to be understood as insignificant. The duration cut-off at 2 months is a fourth example of application of a threshold, in this case involving time. The 2-month requirement for remission is not present in ICD-11 (2).

## 3. Implications of defining episodes using multiple thresholds

**Figure 1:**
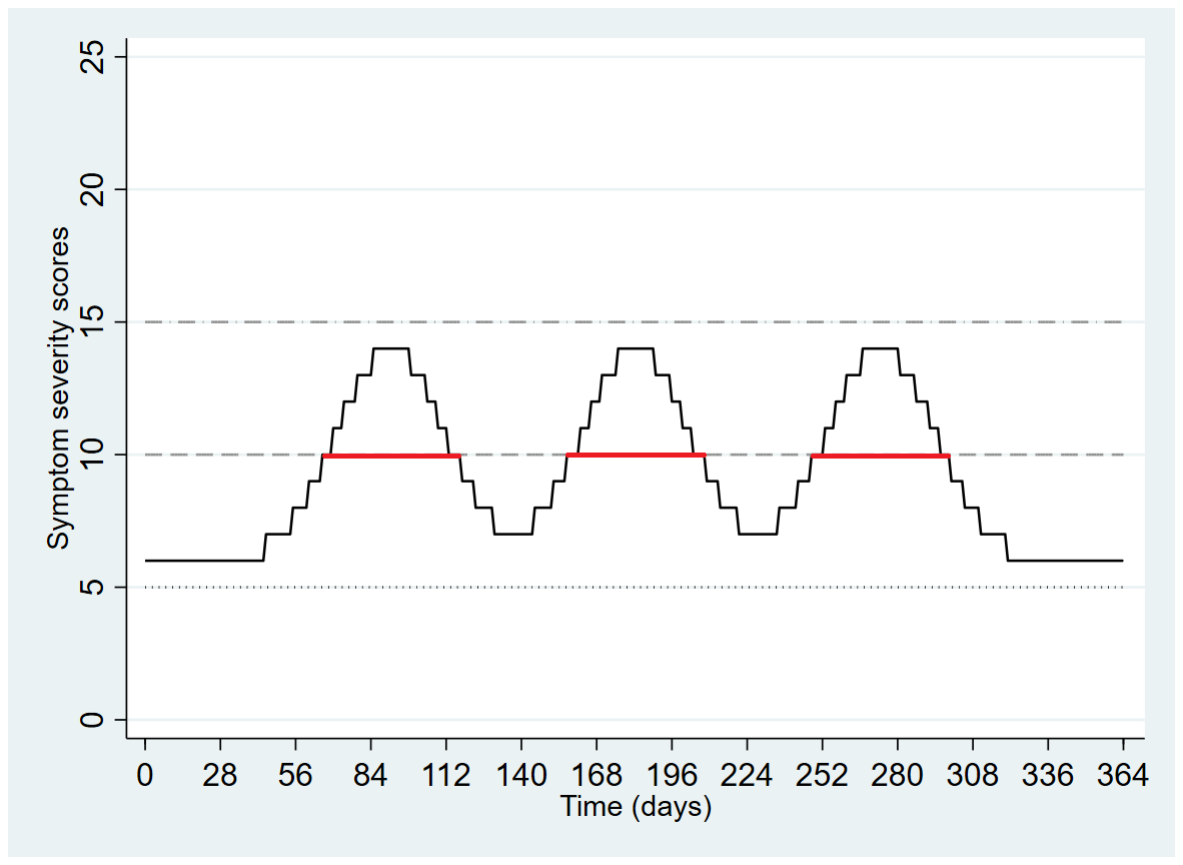
Hypothetical pattern of symptom severity over one year, with application of three symptom severity thresholds.

Figure 1 depicts a hypothetical person’s pattern of depressive symptoms over time. There is a low level of symptoms at baseline followed by three temporary increases in symptoms. The values on the y-axis are arbitrary but have been scaled to a range of scores roughly designed to resemble those of the PHQ-9. The symptom levels during an episode are depicted as normal distributions with rounding to reflect discrete values provided by ordinal scales such as the PHQ-9. The x-axis is scaled to represent 1 year. Examining Figure 1 begins to show how the application of symptom-severity thresholds affects measurement. If a threshold of 15 is used to define entry into an episode, no episodes occur. If 5 is used as a threshold, then there is one protracted episode, lasting the entire year. If a threshold of 10 is used there are three episodes observed, each lasting about 7 weeks, depicted in the Figure using horizontal red lines.

The episodes that emerge from the middle threshold in Figure 1 do not seem problematic. Fluctuating symptoms, as depicted, present the appearance of three episodes when a seemingly suitable threshold is selected. Incidentally, Figure 1 also illustrates the weakness of a purely dimensional approach based entirely on symptom severity at a point in time. Depending on the point at time at which measurement occurred, a symptom severity scale would yield very different results. For example, at day 90, the score is 14 whereas at day 140, it is 7. This is one reason why a simplistic dimensional approach based entirely on symptom severity does not meet the needs of diagnostic assessment.

**Figure 2:**
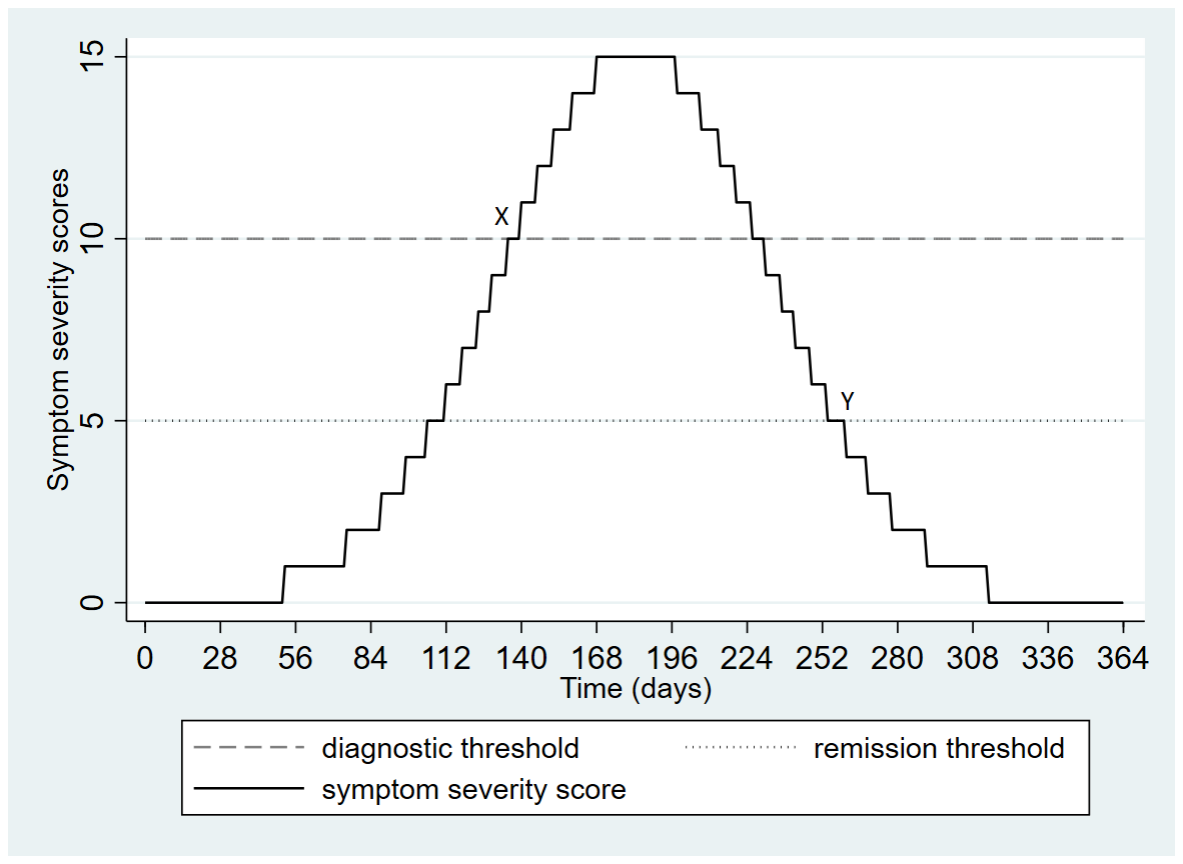
Hypothetical pattern of symptom severity over one year, depicting the onset and remission of a single episode.

In Figure 2, there is an increase in symptoms from an initial level of zero to a peak value at mid-year. Subsequently, the symptom ratings diminish again to zero. The dashed horizontal line on Figure 2 depicts a threshold of severity required by diagnostic criteria. Symptoms exceed this threshold on day 135 and fall below the same threshold on day 231, producing an episode duration of 96 days. However, remission of an episode, both in DSM-5 and ICD-11, requires that there be no significant symptoms. This scenario is depicted in Figure 2, which uses a symptom severity score of 5 as a threshold for “no significant symptoms” depicted by a dotted line. Considered this way, the symptoms exceed the diagnostic threshold on day 135 (as before) but now fall below the remission threshold at day 261 such that the duration of the episode becomes 126 days. In Figure 2, the onset of the episode is labeled X and the time of remission is labeled Y.

**Figure 3:**
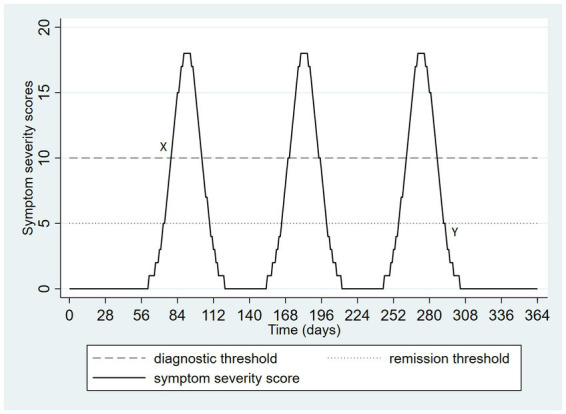
Hypothetical pattern of symptom severity over one year, depicting a potential divergence of DSM-5 and ICD-11 categorization.

In clinical practice, depressive symptoms tend to vary over time, following more complex trajectories than those depicted in Figures 1, 2. Consequently, the impact of application of multiple thresholds becomes even more pronounced. Figure 3 depicts an example. There are three similar peaks of depressive symptoms during a 1-year period. If a diagnostic threshold of 10 is used (dashed horizontal line) and a remission threshold of 5 (dotted horizontal line) then there are three depressive episodes, each lasting about 1 month. This interpretation is consistent with the ICD-11 definition. DSM-5’s remission requirement for at least 2 months without significant symptoms creates a single episode lasting 214 days, poorly characterizing the depicted pattern of symptoms. The starting point for this single lengthy episode is depicted as X and the endpoint is labeled Y. Figure 3 illustrates that the familiar loss of information that occurs with application of a threshold, in this case, it causes a very different categorizations of the same symptom pattern depending on the threshold selected, and varying dramatically on either side of the threshold, as always occurs when episodes are defined in terms of thresholds. If the valleys between the peaks in Figure 3 were to become slightly wider, the single long episode arising from the DSM-5 definition would abruptly, at the threshold of 2 months, become three discrete episodes again. Recommendations for long-term treatment found in guidelines are often based on the number of past episodes. The example, the occurrence of three or more past episodes has been used as a guideline for when maintenance antidepressant treatment is necessary (12). Consequently, the problematic behavior of episode-based definitions assumes increased clinical significance. In the example of Figure 3, a patient with longer gaps between worsening of symptoms may paradoxically become a stronger candidate for long-term maintenance treatment.

**Figure 4:**
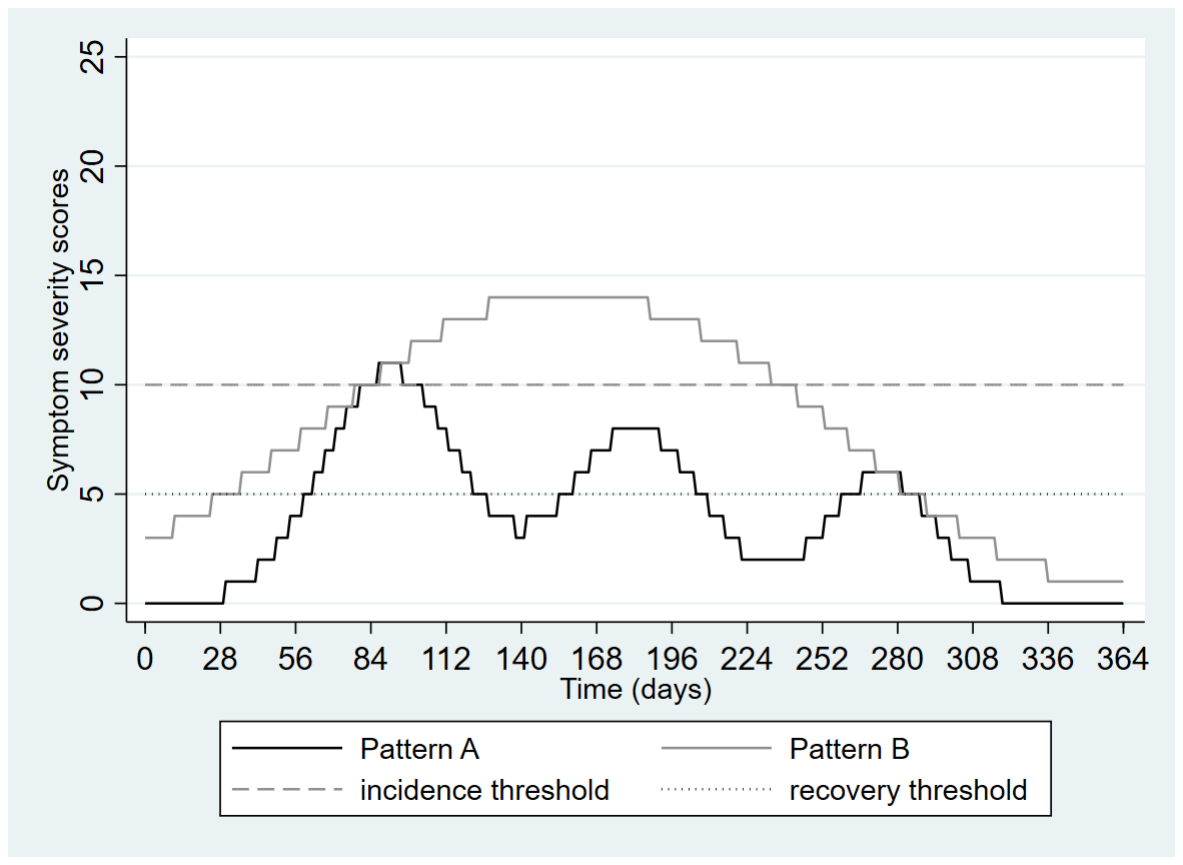
Hypothetical pattern of symptom severity over one year, depicting a loss of information due to categorization using thresholds: different patterns are made to seem similar.

The next two figures depict two patterns of depressive symptoms on the same graph. In Figure 4, the patterns differ; with pattern A always having lower scores than pattern B. Pattern A drops below the threshold for remission on two occasions but not long enough to meet the duration threshold for remission in DSM-5. Although the patterns of symptoms are very different, both patterns have the same onset date (day 79) and remission date (day 292), and an identical duration (30 weeks, with application of the DSM-5 remission definition). This example presents a familiar problem with categorization of dimensions: different situations may be categorized in ways that cause them to appear similar.

**Figure 5:**
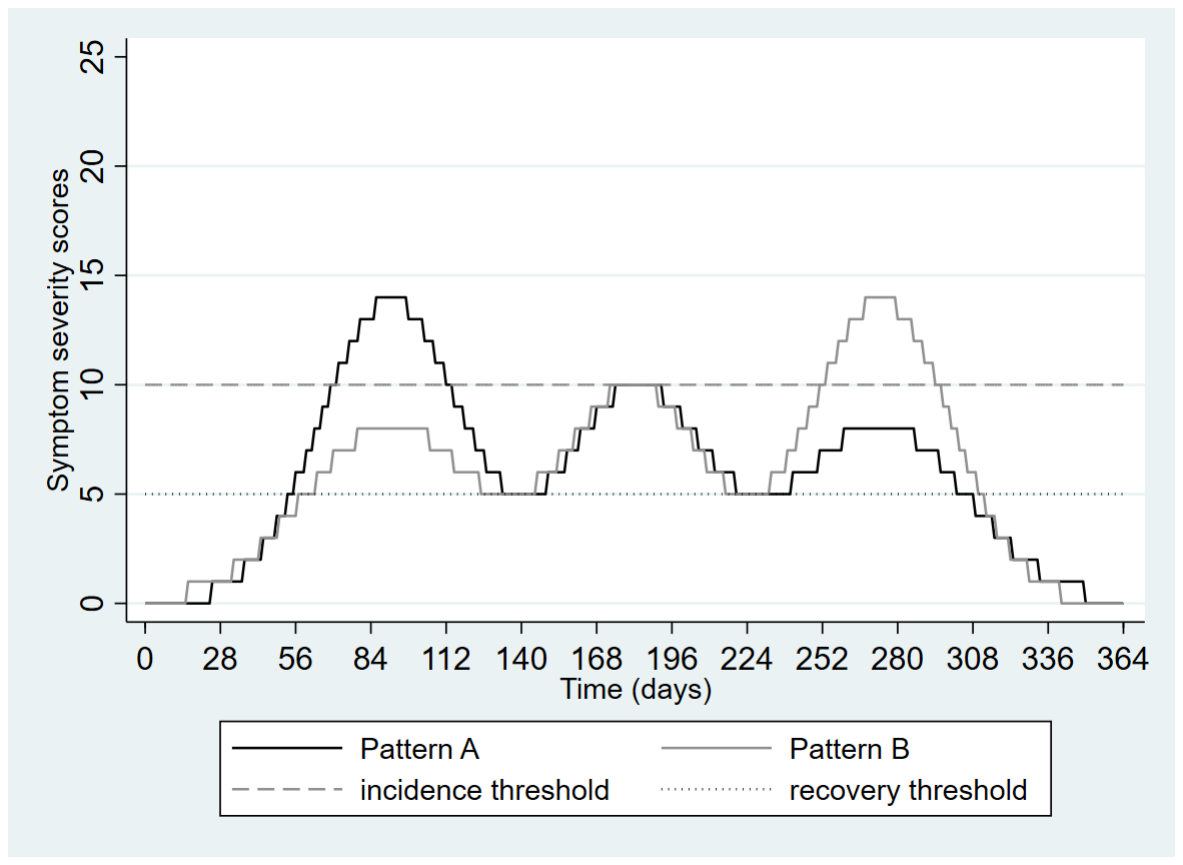
Hypothetical pattern of symptom severity over one year, depicting a loss of information due to categorization using thresholds: similar patterns are made to seem different.

Figure 5 again shows two differing patterns of depressive symptoms. In pattern A, there is an initial higher peak followed by two smaller peaks whereas in pattern B there are smaller peaks preceding a larger one. However, the overall burden and severity of the symptoms are identical. The only difference is the ordering of the peaks in the symptom patterns. Despite this, application of the DSM-5 definition results in two very different categorizations. Pattern A results in a single episode starting on day 69 and resolving on day 310, therefore lasting 241 days or approximately 34 weeks. In contrast, pattern B results in a single episode lasting only 57 days or about 8 weeks, approximately the amount of time required for a full trial of an antidepressant medication. Figure 5 shows the “flip side” of the threshold-based categorization issue. Very similar patterns can be made to appear very different.

**Figure 6:**
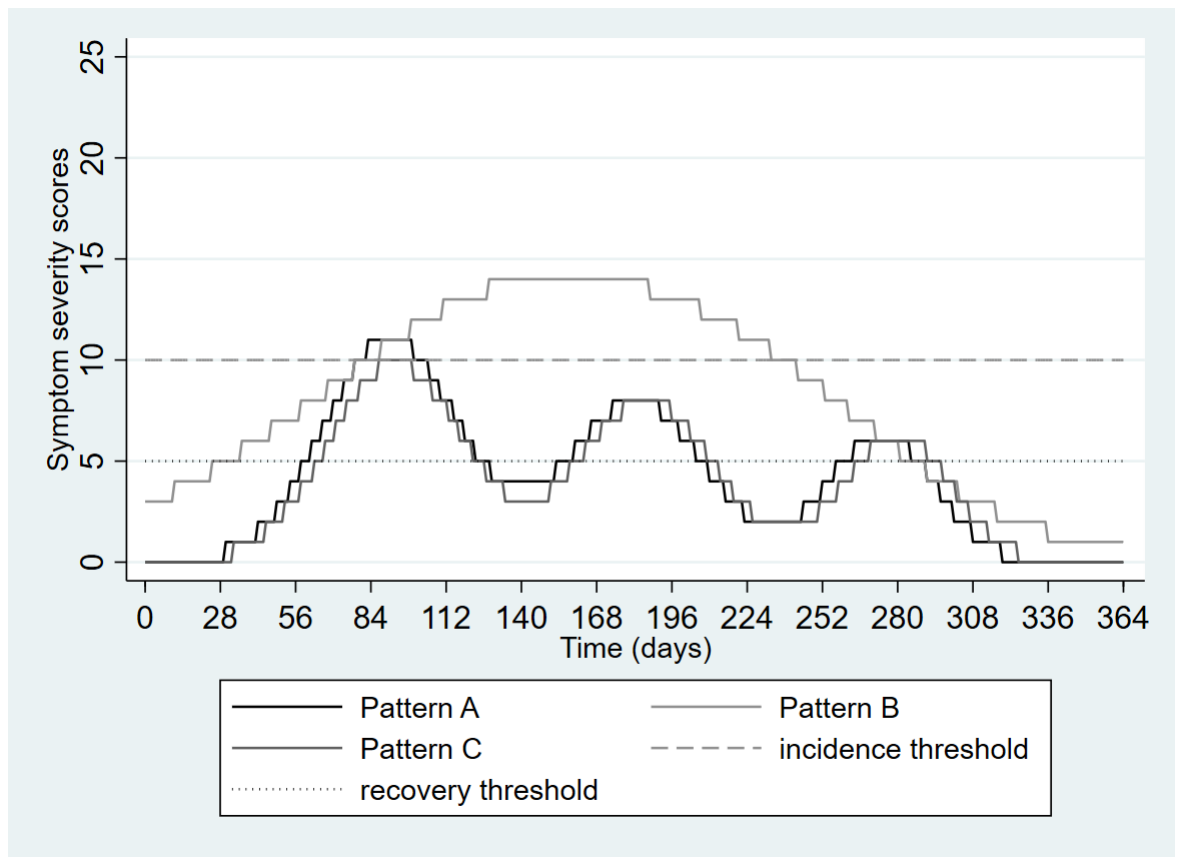
Hypothetical pattern of symptom severity over one year, depicting a loss of information due to categorization using thresholds: similar patterns are made to seem different and vice versa.

These graphics presented above illustrate the loss of information that can occur with application of a one threshold out of the four required to construct an episode in DSM-5. Figure 6 extends Figure 4 by adding a third pattern, nearly identical to pattern A except that the duration of time in which the incidence threshold is exceeded is now only 13 days. This pattern is labeled pattern C in Figure 6. This very small change means that no episode occurred at all, despite this pattern being nearly identical to pattern A, which itself problematically appeared nearly identical to a more prolonged and severe episode (pattern B).

## 4. Attempts to add dimensionality to the diagnosis of depression

As the drawbacks of categorization are well known, several proposals have been made to integrate dimensional measurement into the diagnostic process. For example, based on an analysis of data from a cohort study conducted in Zurich between 1979 and 1993, Angst & Merikangas noted that dimensional constructs such as maximal symptom counts, duration of episodes, and frequency of episodes correlated with several characteristics (e.g., family history) traditionally regarded as validators of diagnostic definitions of depression (13). These observations were interpreted as validation of a multi-dimensional model. However, the multidimensional concept remained episode-based and is therefore subject to threshold-related ambiguities, examples of which were described above. For example, the dimension of episode frequency was assessed over a one-year period on a scale ranging from 1 episode per year to “daily” episodes. In practice, attempts to distinguish discrete daily episodes from longer episodes characterized by daily symptoms require decisions to be made about when episodes start and end. Both longer episodes and more frequent episodes were associated with the validity indicators, yet a person with the maximal episode duration of one year can only have the lowest possible non-zero episode frequency of 1 per year. Such problems with multidimensional approaches have not been resolved. In a 2005 commentary, Brown & Barlow (14) observed that “no strong proposals have emerged with regard to exactly how dimensional classification could be introduced in the DSM.” Adopting Goldberg’s Platonic view, they argued that etiologically informed dimensional models (arising either from psychological or neuroscience research) would eventually solve the problems of categorization. In the meantime, they argued that an acceptable compromise would be to add dimensional measures to the categories defined in the (then) upcoming DSM-5 (14). In 2008 Klein (15) proposed a two-dimensional approach to classification of depression, with the two dimensions being severity and chronicity. However, the proposed strategy remained rooted in the concept of an episode. For example, the chronicity scale was based on statements about episodes, e.g., “single episode of ≤2 years duration” was one of four proposed levels of chronicity (15). This is another example of a dimensional proposal that remains rooted in the idea of episodes. In a 2019 review, Ruscio (16) proposed a “multiple threshold” model. Rather than adopting a single threshold, several meaningful thresholds could be applied along a continuum, creating a potentially useful balance of utility and validity across the broad spectrum of manifestations of depression (16). The challenge of this approach, however, lies in the nature of the dimension being categorized. There needs to be a single dimension upon which multiple thresholds can be applied. This would require integration of information about multiple dimensional characteristics (such as those proposed in Angst & Marikangas’ multi-dimensional model). A potential solution to this problem is described in Section 6 of this manuscript.

## 5. Partial solutions to the problems created by threshold-defined episodes

One partial solution to these problems may be found in the way that DSM-5 has sought to create a more dimensional perspective by defining mild, moderate, and severe descriptors to major depressive episodes, consistent with Ruscio’s (16) recommendation for multiple thresholds, except that DSM-5 does not address depression falling below the DSM-5 threshold for MDE. However, in DSM-5, multiple thresholds cannot be applied until after an episode has been identified. Application of such specifiers creates a more dimensional description of an episode but does not address the issues caused by application of thresholds in the construction of episodes in the first place.

Another partial solution is to accept the flaws of the current definitions but allows for their correction by strongly prioritizing clinical judgment above the classification formed by diagnostic criteria. This approach aligns with the idea that diagnostic criteria provide an imperfect but simple approach to classification, better regarded as an indicator of health status rather than as a diagnosis *per se*. In keeping with contemporary practice, the severity, persistence, dangerousness, and functional impact of depression (all integrated through clinical judgment) may be a more useful guide to clinical action than a criterion and episode-based classification.

Technology may also help. If depression is to be measured without reference to episodes, this cannot be based on deciding when depression starts or ends but must rather be based on quantifying the extent of depression during a specified time interval. Symptom rating scales do exactly this, but usually only over a brief time periods such as the past week or two weeks. The average severity of depressive symptoms over longer and more clinically salient intervals could be determined by collecting weekly or monthly symptom ratings using mobile apps, mood trackers, or automated survey functions of database software such as REDCap, providing a profile of symptoms over time, similar perhaps to what is depicted in the Figures above. Considering Figure 4, the average daily score for Pattern A is 8.3 whereas that for Pattern B is 4.1, avoiding the artificial similarity imposed on these different patterns by the DSM-5 and ICD-11 definitions. Performing the same calculation on the Figure 5 data yields an average of 5.9 in both groups, illustrating how this approach can avoid the emergence of artificial or exaggerated differences as a result of episode construction.

A facsimile of the approach of measuring average scale scores over time may be generated using information collected in clinical interviews and may therefore be easier to implement in clinical practice. For example, inquiries could be aimed at determining the amount of time during an interval (such as the number of weeks in the past year) that was spent by a patient at different levels of depression such as: (1) no depression, (2) very mild depression, (3) mild depression, (4) moderate depression, (5) severe, or (6) very severe depression. On an ordinal scale of this sort, each patient will have a median value – a value for which 50% of, days or weeks during the past year, fall below and 50% fall above that category. This creates a dimensional metric that is free of thresholds and episodes. The approach resembles Ruscio’s multiple threshold strategy (16), except that it starts with a time frame of interest rather than a predefined episode of depression.

## 6. A proposed dimensional metric that is not dependent on defining episodes

The clinical approach described above leaves the integration of characteristics such as symptom severity and functional impairment to the clinical interview process, which is one way of avoiding the cumbersome and problematic nature of a multidimensional approach. For clinicians and researchers interested in more formalized quantification there may be other opportunities to consider. For example, the Global Burden of Disease project has used standard gamble methodologies to ascribe disability weights, which quantify health loss in over 300 diseases and disabilities (17). The concepts of health loss or health utility are a more formal way to integrate various dimensions of the experience of health issues such as depression onto a single dimension, with a weight of zero representing no health loss and 1.0 representing a state similar to death. The Global Burden of Disease project reports three vignettes representing depressed states and has conducted research to formally assign a disability weight to each state. These weights reflect the following states:

- Mild: “feels persistent sadness and has lost interest in usual activities. The person sometimes sleeps badly, feels tired, or has trouble concentrating but still manages to function in daily life with extra effort.” Weight: 0·145
- Moderate: “has constant sadness and has lost interest in usual activities. The person has some difficulty in daily life, sleeps badly, has trouble concentrating, and sometimes thinks about harming himself (or herself).” Weight: 0·396
- Severe: “has overwhelming, constant sadness and cannot function in daily life. The person sometimes loses touch with reality and wants to harm or kill himself (or herself).” Weight: 0·658

Presenting such, or ultimately more sophisticated, scenarios to patients while obtaining (by history) an estimated proportion of time spent in each state over a meaningful time interval, one can calculate the sum of products of these proportions and their associated weights, creating a dimensional metric. For example, a patient spending 6 months of the past year mildly depressed and 6 months moderately depressed can be assigned an average level of health loss over the year of (0.5 × 0.145) + (0.5 × 0.396) = 0.27. This calculation places the assessment immediately onto an episode-free dimensional scale. Tables 1, 2 consider the same scenarios graphed in Figures 4, 5. Assuming (solely for the purpose of illustration) that the traditional PHQ-9 interpretive ranges mapped onto the health states associated with the disability weights describe above (<5 = none, 5–9 = mild, 10–19 = moderate/moderately severe, 20–27 = severe), Tables 1, 2 present a calculation of this dimensional measure for the 365 days depicted in these two Figures. Here the advantages of the dimensional approach are illustrated by the distinctiveness of the measurements from Figure 4, in contrast to the episode-based approach, and the equivalence of the estimates from Figure 5, again in contrast to the episode-based approach. Pattern C in Table 1 further illustrates the value of dimensional assessment. The trivial difference between Pattern A and C in Figure 6 is correctly quantified as a trivial difference. In these scenarios, the strategy of weighting time spent in various severity states outperforms the strategy of constructing episodes by application of thresholds.

**Table 1:** Illustrative example of using health states and disability weights to construct a dimensional depression metric, data from Figures 4, 6.

Severity level	Proportion of days at each severity level	Disability weight	Product of proportion and weight
Pattern A			
None	0.56	0.000	0.000
Mild	0.37	0.145	0.054
Moderate	0.08	0.396	0.032
Severe	0.00	0.658	0.000
Total dimensional score			0.085
Pattern B			
None	0.271	0.000	0.000
Mild	0.277	0.145	0.040
Moderate	0.452	0.396	0.179
Severe	0.000	0.658	0.000
Total dimensional score			0.219
Pattern C – from Figure 6			
None	0.581	0.000	0.000
Mild	0.384	0.145	0.056
Moderate	0.036	0.396	0.014
Severe	0.000	0.658	0.000
Total dimensional score			0.070

**Table 2:** Illustrative example of using health states and disability weights to construct a dimensional depression metric, data from Figure 5.

Severity level	Proportion of days at each severity level	Disability weight	Product of proportion and weight
Pattern A			
None	0.299	0.000	0.000
Mild	0.545	0.145	0.079
Moderate	0.156	0.396	0.062
Severe	0.000	0.658	0.000
Total dimensional score			0.141
Pattern B			
None	0.299	0.000	0.000
Mild	0.545	0.145	0.079
Moderate	0.156	0.396	0.062
Severe	0.000	0.658	0.000
Total dimensional score			0.141

A potential criticism of the approach depicted in Tables 1, 2 is that they do not entirely escape the issue of categorization, since they depend on classifying symptom severity and the associated weighting into categories. This is certainly true, but breaking the binary category into a series of subgroups is a step toward improved accuracy, similar to Ruscio’s multiple threshold model (16). While the quantification in Tables 1, 2 is presented for purely illustrative purposes, it helps to identify an approach by which dimensional measures could be developed and validated in research applications, and as an approach to dimensional diagnostic assessment for clinical practice.

If research and practice are to improve, it may be necessary to jettison an approach to diagnosis that is based on the identification of episodes. Even in situations in which symptoms fluctuate from baseline levels, as depicted in the Figures above, defining episodes through application of thresholds is vulnerable to problems of classification. One may hypothesize that a dimension approach that is free from the problems of episode classification will reduce misclassification, provide improved prognostic prediction, and therefore improve support for clinical decision-making.

## Data availability statement

The original contributions presented in the study are included in the article/supplementary material, further inquiries can be directed to the corresponding author.

## Author contributions

The author confirms being the sole contributor of this work and has approved it for publication.

## Conflict of interest

The author declares that the research was conducted in the absence of any commercial or financial relationships that could be construed as a potential conflict of interest.

## Publisher’s note

All claims expressed in this article are solely those of the authors and do not necessarily represent those of their affiliated organizations, or those of the publisher, the editors and the reviewers. Any product that may be evaluated in this article, or claim that may be made by its manufacturer, is not guaranteed or endorsed by the publisher.
